# Implementation determinants and mechanisms for the prevention and treatment of adolescent HIV in sub-Saharan Africa: concept mapping of the NIH Fogarty International Center Adolescent HIV Implementation Science Alliance (AHISA) initiative

**DOI:** 10.1186/s43058-021-00156-3

**Published:** 2021-05-22

**Authors:** Gregory A. Aarons, Kendal Reeder, Nadia A. Sam-Agudu, Susan Vorkoper, Rachel Sturke

**Affiliations:** 1 Department of Psychiatry, University of California San Diego, 9500 Gilman Drive (0812), La Jolla, CA 92093-0812 USA; 2 UC San Diego Dissemination and Implementation Science Center (UC San Diego ACTRI DISC), Altman Clinical and Translational Research Institute, La Jolla, CA 92093 USA; 3 Child and Adolescent Services Research Center, 3665 Kearny Villa Rd., Suite 200N, San Diego, CA 92123 USA; 4 Institute of Human Virology and Department of Pediatrics, University of Maryland School of Medicine, 725 West Lombard Street, Baltimore, MD 21201 USA; 5 International Research Center of Excellence, Institute of Human Virology Nigeria, Plot 252 Herbert Macaulay Way, Abuja, Nigeria; 6 NIH Fogarty International Center, Center for Global Health Studies, Bethesda, MD USA

**Keywords:** Concept mapping, Implementation science, Sustainment, EPIS framework, Adolescent, HIV, Africa

## Abstract

**Introduction:**

Adolescent HIV prevention and treatment is a high priority for youth healthcare in sub-Saharan Africa.

**Methods:**

This study employed concept mapping to identify factors that impact the implementation of HIV prevention and intervention programs for adolescents in sub-Saharan Africa. Key stakeholders including researchers, policymakers, and non-governmental organization (NGO) personnel constituting membership of the NIH-sponsored Adolescent HIV Prevention and Treatment Implementation Science Alliance responded to the question: “In your experience, what factors have facilitated or hindered implementation of evidence-based HIV prevention or treatment for adolescents in sub-Saharan Africa?” Participants generated statements in response to the focus question, sorted them into thematically relevant groups, and rated each statement on its importance and changeability.

**Results:**

Through data analyses and participant feedback, 15 distinct themes were derived. “Workforce/Workflow” and “HIV Stigma and Adolescent Development” were rated highest for importance, and “Threshold Conditions for Treatment” and “Structure of Implementation Efforts” were rated most changeable.

**Conclusions:**

Understanding implementation science determinants and mechanisms can facilitate the uptake of successful implementation and sustainment strategies for the prevention and treatment of HIV in a given context. We placed determinants and mechanisms within the Exploration, Preparation, Implementation, Sustainment (EPIS) framework to provide greater contextual integration with broader theories in implementation science. Implementers across multiple disciplines can use these findings to improve the scale-up of evidence-based practices for adolescent HIV prevention and treatment in sub-Saharan Africa. Implementation approaches that consider the determinants and mechanisms identified in this study and integrated in implementation frameworks will likely have utility for other health conditions and contexts.

**Supplementary Information:**

The online version contains supplementary material available at 10.1186/s43058-021-00156-3.

Contributions to the literature

- This study identified multiple factors likely to act as implementation determinants and mechanisms for HIV prevention and treatment for adolescents in sub-Saharan Africa.
- Work focused on understanding implementation determinants and mechanisms should build on implementation frameworks and advance implementation science.
- This study also illustrates how concept mapping results for implementation can be placed within the Exploration, Preparation, Implementation, Sustainment (EPIS) implementation framework’s constructs and processes.
- The findings presented here can guide the development and/or tailoring of implementation strategies for adolescent HIV prevention and treatment in sub-Saharan Africa.
- Findings from this study can inform future implementation science studies related to HIV prevention and treatment of HIV among adolescents.

## Background

Implementation and sustainment of evidence-based practices (EBPs) is a challenge, even for established and relatively well-funded healthcare systems in high-income countries. Implementation of EBPs in low- and middle-income countries presents challenges that may be common across settings (e.g., poor healthcare financing and limited human resources), but unique with respect to the disease being addressed, the country or region of implementation, and the target population. Over the last 10–15 years, there has been increased interest and research in implementation science (IS) to accelerate the translation of research into practice. There is growing emphasis on IS for adolescent HIV prevention and treatment, as evidenced by the Adolescent Medicine Trials Network for HIV/AIDs in the USA [1, 2] and the International Epidemiology Databases to Evaluate AIDS [3].

The challenges of implementing EBPs for adolescent HIV prevention and treatment are not unique; the benefits of proven interventions have not been fully realized because of enduring barriers to uptake, replication, and scale-up in many settings. To better address “knowledge-practice” gaps, more emphasis is being placed on theoretical processes and mechanisms that underpin effective dissemination and implementation. In alignment with this, and understanding healthcare needs particular to adolescents, there is an increasing focus on IS in adolescent HIV. The IS field holds promise for addressing critical challenges that limit successful implementation of EBPs for this population. An IS approach to substantial advancements is to have deliberate and strategic efforts to facilitate collaboration, communication, and relationship-building among the generators and users of research evidence.

To advance IS and address challenges to effective implementation of EBPs for adolescent HIV in sub-Saharan Africa, the National Institutes of Health’s Fogarty International Center created the Adolescent HIV Prevention and Treatment Implementation Science Alliance (AHISA) [4–6]. The overarching objective of the AHISA platform is to facilitate better utilization of scientific evidence in adolescent HIV programming, while simultaneously helping to ensure that research is country-driven and responsive to the adolescents’ health needs. AHISA comprises teams of implementation scientists and their in-country implementing partners from 11 sub-Saharan African countries. This innovative platform provides opportunities for enhanced communication and collaboration between implementation scientists, program implementers, and policymakers in achieving its goals. Through this approach, AHISA thereby promotes scalable, empowering, and sustainable interventions.

AHISA and other implementation science communities have recognized the need for better understanding of determinants (i.e., predictors), mechanisms (i.e., mediators/moderators), and targets (i.e., outcomes) in IS studies [6–8]. It is also important to consider and address how implementation mechanisms can affect the implementation process and attenuate or potentiate the effect of determinants on implementation outcomes [9]. To increase understanding of the effects of determinants and mechanisms, it is also necessary to identify relevant implementation outcomes for a specific target population and in a given context [10]. To do so, it is critical to garner knowledge based on the experiences of implementers familiar with the patient or public health population, the context, and other relevant stakeholders who may be important in implementation and sustainment. This information is also necessary for operationalizing key constructs that may be important in implementation efforts and research projects within specific contexts [11].

A key next step is to map this information to implementation conceptual frameworks to support and drive hypothesis formulation and IS project design. To this end, we place this work in the context of the Exploration, Preparation, Implementation, Sustainment (EPIS) framework with is both a process and determinant framework [11, 12]. The EPIS framework has been used in multiple studies in sub-Saharan Africa (e.g., Nigeria, Kenya, Sierra Leone) [13–15]. The EPIS framework is particularly useful in this context due to its broad dimensions such as outer system context (e.g., sociopolitical constraints, ministries of health), inner context (e.g., service organizations such as non-governmental organizations), bridging factors (e.g., relational ties, formal arrangements, capital exchange, processes) that link outer and inner contexts [16, 17], characteristics of practices to be implemented, and interorganizational relationships within and between outer and inner contexts [11]. As will be described in the “Methods” section, “bridging factors” in EPIS are distinct from “bridging values” in concept mapping (i.e., conceptual overlap in derived clusters) [18, 19].

## Methods

### Aims

The present study was initiated to accomplish two aims. The first aim was to identify factors that act as determinants and mechanisms likely to affect the implementation and sustainment of evidence-based HIV prevention and treatment programs for adolescents in sub-Saharan Africa. The second aim was to frame the results in a highly cited and utilized IS framework, the EPIS implementation framework in order to elucidate how identified determinants and mechanisms may be relevant across implementation phases, and in considering factors both within and bridging the outer (system) and inner (organizational and/or community) [11, 12].

### Design

This study employed concept mapping (CM), a mixed-methods approach to data collection and analysis, to capture a range of stakeholder perspectives from within the AHISA network [18]. Since CM incorporates input from relevant stakeholders, the approach is apt in determining crucial themes and action items, facilitating dialogue between stakeholder groups (e.g., researchers, non-governmental organizations), and has high utility for implementation research and practice [20, 21]. We used an inclusive and participatory mixed-methods approach to identify potential determinants and mechanisms likely to be important in the implementation of EBPs for adolescents living with HIV in sub-Saharan Africa [9, 22]. We considered this specific population (i.e., adolescents) within the context of multiple countries represented in the AHISA network to identify relevant implementation factors that would be generalizable across countries.

### Participants

Forty-five AHISA member-stakeholders participated in at least one phase of the CM activity through the virtual Concept Systems, Inc. interface. As shown in Table 1, participants represented a range of roles, disciplines, and countries. Most participants had a primary role related to research (80%). The mean number of years of experience in implementation science was 6.2 years (range=0.5–25.0 years), and the mean number of years in adolescent HIV programming/research was 10.3 years (range=0.9–24.0 years). This study was approved by the Institutional Review Board of the University of California, San Diego as exempt research.

**Table 1:** Participant characteristics

	Number	Percentage
Primary role setting		
Research	36	80%
NGO	8	18%
Policy	1	2%
Primary discipline		
Medicine	22	49%
Social/behavioral science	8	18%
Public health	7	16%
Psychology	3	7%
Nursing	2	4%
Social work	2	4%
Others: monitoring and evaluation	1	2%
Country of focus		
Kenya	10	22%
South Africa	8	18%
Nigeria	6	13%
Tanzania	5	11%
Zimbabwe	5	11%
Uganda	3	7%
Botswana	2	4%
Ghana	2	4%
Rwanda	2	4%
Zambia	2	4%
Malawi	1	2%
Average years of experience in implementation science	Average years of experience in adolescent HIV	
6.23	10.33	

### Concept mapping approach

The CM approach involves six phases: (1) *preparation*, in which researchers identify stakeholder participants and work collaboratively with them to create a focus question; (2) *generation (or brainstorming)*, during which participants produce responses (i.e., statements) to the focal question; (3) *structuring (or sorting and rating)*, during which each participant sorts the entire pool of statements based on thematic similarity and then rates each statement based on a priori dimensions (i.e., importance and changeability); (4) *representation*, in which researchers utilize multidimensional scaling (MDS) and conduct cluster analyses to generate a “concept map” displaying statements spatially based on how they were sorted (i.e., statements more frequently sorted together are displayed in closer proximity); (5) *interpretation*, during which researchers and participants work together to label and interpret clusters of statements; and (6) *utilization*, in which researchers and participants collaboratively identify action items and next steps based on the concept map. The method allowed for stakeholders to process through these phases without direction from facilitators so that relevant factors may emerge with minimal external influence and bias. We describe below how we applied the CM approach.

The preparation and generation process began with stakeholder engagement and the development of the focus question: “In your experience, what factors have facilitated or hindered implementation of evidence-based HIV prevention or treatment for adolescents in sub-Saharan Africa?” The focal question was generated with 88 stakeholders, including AHISA members and invited guests-members of the Uganda health ministry, US state department, and Ugandan researchers and implementers partnering with some of the AHISA members.

The in-person AHISA meeting was held in February 2019 in Kampala, Uganda. Group brainstorming began at the in-person meeting, and additional statements from 17 participants were gathered virtually using the Concept Systems, Inc. interface. Statements were reviewed and duplicate statements were removed. The process resulted in 102 unique statements regarding factors likely to facilitate or hinder implementation.

In the structuring phase, 75 AHISA members were invited through the Concept Systems, Inc. software to virtually sort the 102 statements into categories according to their views and based on similarity of the statements’ meaning or theme. Participants were asked to name each group of statements in a way that described its theme or contents. Invited members participated in the sorting phase by rating each statement on two dimensions: “importance” and “changeability,” using a 6-point Likert scale (0=not at all; 5=to a very great extent). Participants were prompted with the following: “How important is this for the implementation of evidence-based HIV prevention or treatment for adolescents in sub-Saharan Africa?” and “How changeable is this?” Thirty-four (45%) of the 75 invited stakeholders provided ratings for statements on the importance dimension, and 32 (42.6%) provided ratings on the changeability dimension. Overall, 45 (60%) invited participants participated in at least one CM activity after the initial meeting in Kampala.

**Fig. 1:**
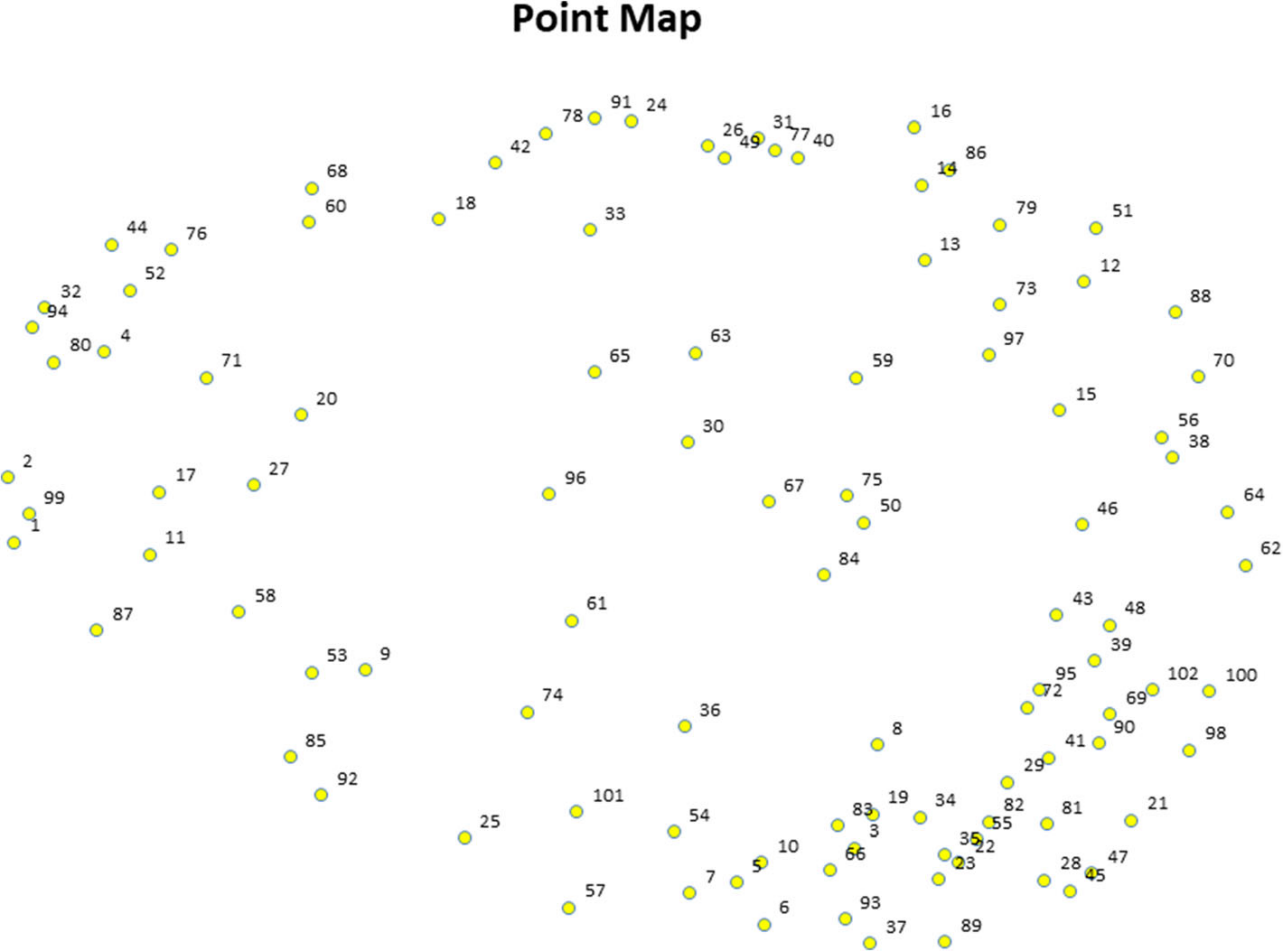
Point Map, with each individual statement represented by a point and labeled with its unique statement number (each statement can be found in Additional File 1).

In the representation phase, Concept Systems, Inc. software was used to analyze statement sorting and rating data. We utilized MDS and hierarchical cluster analysis to analyze the sorted and rated statements. As shown in Fig. 1, the MDS analysis resulted in a Point Map wherein all statements were represented visually as points. These points were placed such that statements that were sorted together more frequently by participants were located in nearer proximity to each other. The fit of the MDS solution is measured with a stress value, with lower values indicating less distortion in distances displayed on the map, and thus better overall fit between the aggregated similarity matrices and the MDS solution [23, 24]. The average stress value for CM across studies has been found to be 0.285, with a range from 0.155 to 0.352 [18, 20]. The solution for the Point Map in this study had a stress value of 0.263 after 13 iterations, thus indicating a satisfactory fit.

The MDS solution also provided information on bridging values, which range from 0 to 1 and indicate the extent to which a statement was sorted similarly by all participants [18]. A bridging value of 0 indicates that a statement was sorted identically, whereas a bridging value of 1 indicates the statement was sorted differently across participants [25]. Statements with high bridging values serve as links between different clusters and may be conceptually related to several statements on the map; therefore, clusters with low bridging values are generally more cohesive and easier to interpret [18, 19]. As with importance and changeability ratings, bridging values are averaged to produce a cluster-level bridging score.

**Fig. 2:**
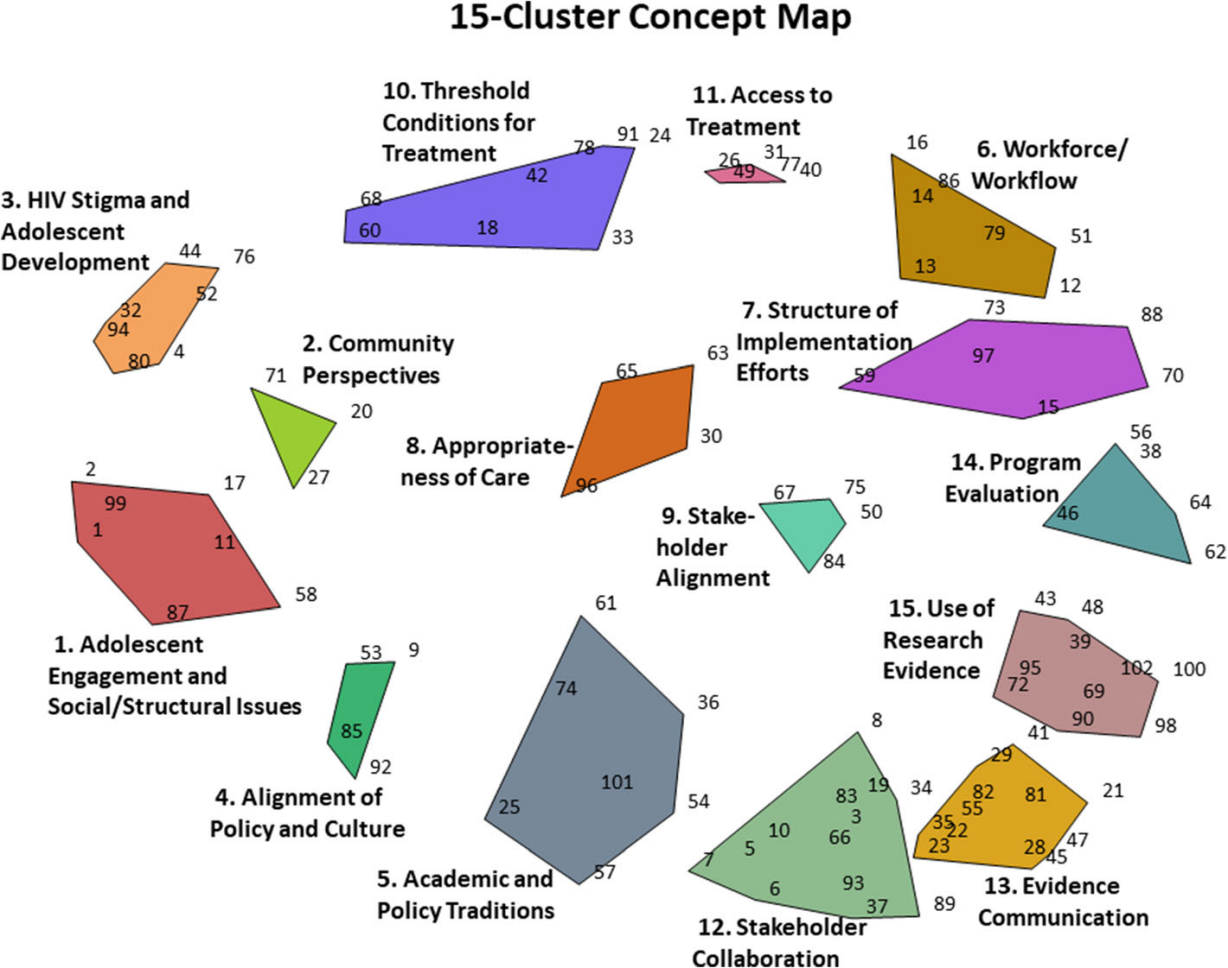
Final 15-cluster Concept Map generated in the representation phase. Cluster labels were created collaboratively between the research team and participants. Individual statements within each cluster are labeled with their corresponding statement number

The Point Map was subsequently used for cluster analysis, which resulted in concept maps that organize statements into thematically distinct clusters based on their proximity. The analysis team (GAA, KR) considered concept maps with varying numbers of clusters, starting with a large number of clusters (i.e., 18) and moving down to determine which map contained the highest number of thematically distinct clusters while retaining parsimony. As shown in Fig. 2, this process resulted in a final 15-cluster Concept cluster map that was determined by the analysis team as the most suitable concept map.

**Fig. 3:**
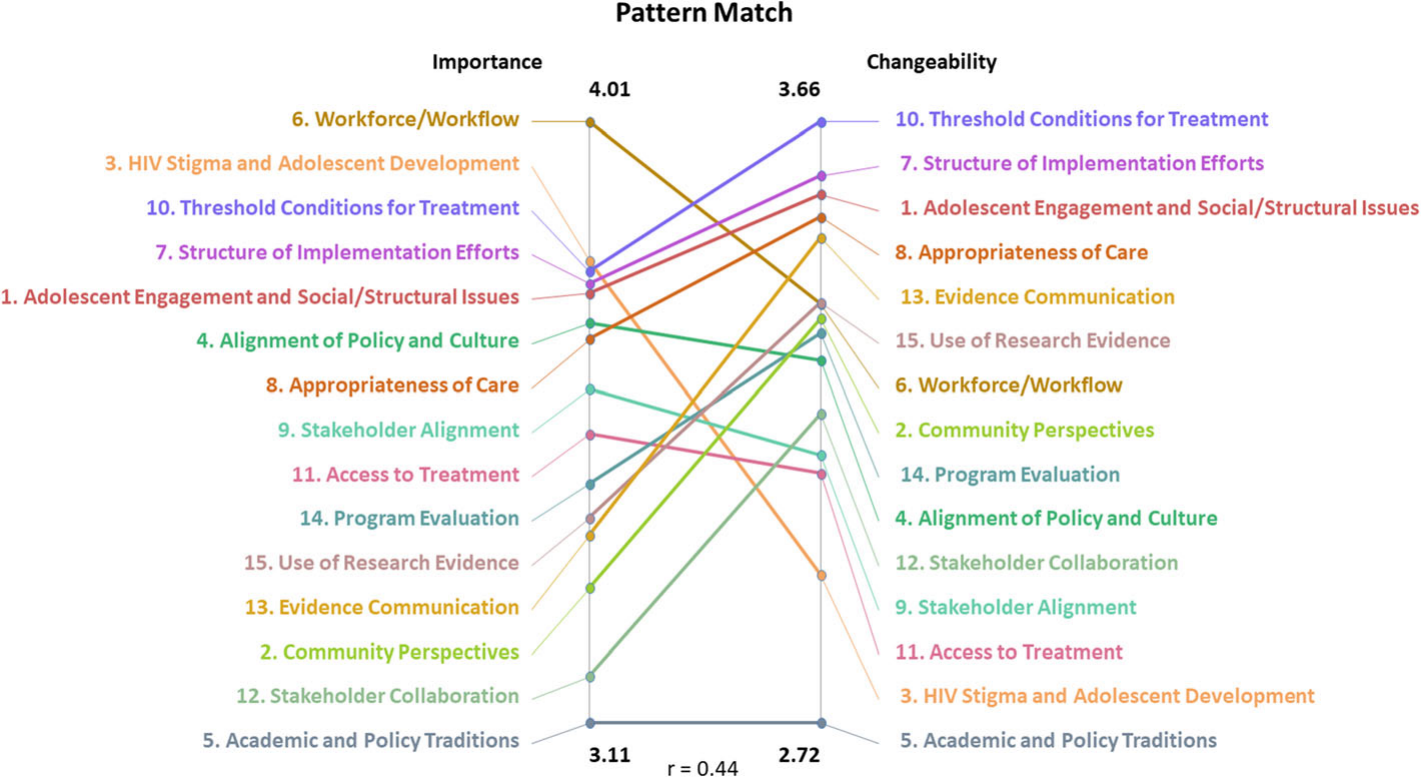
Pattern Match Map showing each cluster plotted by the average importance and changeability ratings of its statements. The overall correlation between the average importance and changeability ratings was *r* = 0.44

**Fig. 4:**
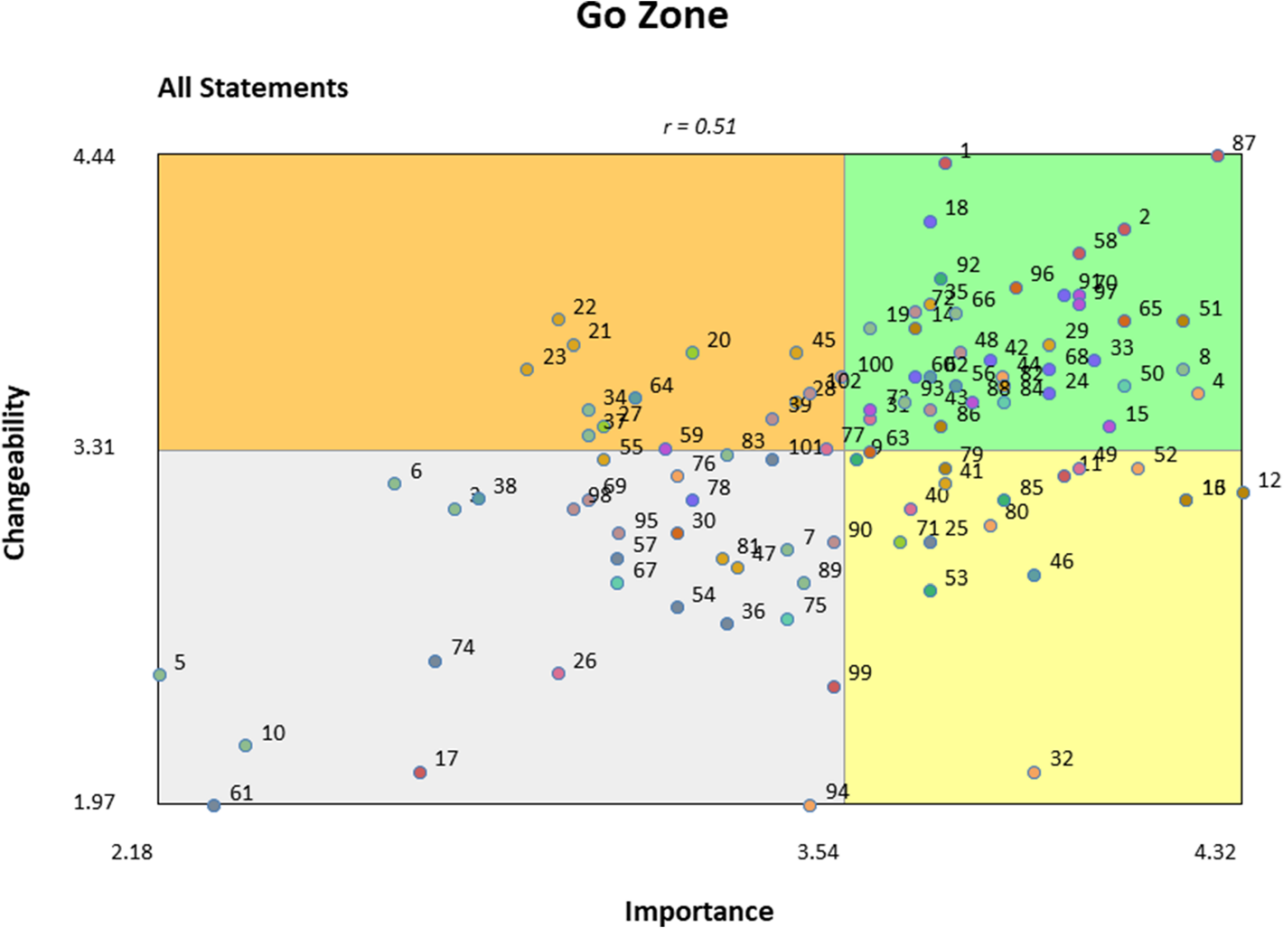
Go Zone map displaying all 102 statements plotted as a function of their average importance and changeability ratings. The “Go Zone” refers to the green quadrant, in which the most highly important and changeable statements are located. The overall correlation of importance and changeability ratings was *r* = 0.51

The preliminary results and the 15-cluster Concept Map were then presented to 57 AHISA members in the interpretation phase during an in-person meeting in Cape Town, South Africa, in February 2020. Participants provided feedback on the results, confirming the 15-cluster Concept Map and collaboratively naming each cluster to best represent the common theme. Following interpretation, participants reviewed Pattern Match and Go Zone Maps (Figs. 3 and 4, respectively). The Pattern Match Map showed the correlation between the average importance and changeability ratings for each cluster, and the Go Zone Map displayed all 102 statements as a function of their importance and changeability rating. To further explore potential action areas while considering the conceptual overlap across clusters, we examined the Go Zone Map (Fig. 4) to investigate the importance and changeability ratings of individual statements. Based on the 15-cluster Concept Map, the Pattern Match Map, and the Go Zone Map, participants collaboratively identified action items and next steps. Participants who partook in interpretation and utilization phases but had not completed the earlier activities were invited to complete sorting and rating virtually afterwards, resulting in two additional responses included in our final results.

## Results

As shown in Fig. 2, the final concept map is comprised of 15 thematically distinct clusters and Table 2 provides detail of the cluster content and rating and ranking of each cluster. Each of the 102 statements within the map is represented by a unique number and statements located more proximally to each other indicate that those statements were more frequently sorted together by participants, while numbers located distally were sorted together less frequently.

**Table 2:** All clusters from the 15-cluster Concept Map, with the average importance and changeability ratings across all statements within the cluster and their relative rank

	Average importance rating within cluster		Average changeability rating within cluster		Combined	
	Cluster rating	Cluster rank	Cluster rating	Cluster rank	Cluster rating	Cluster rank
1. Adolescent engagement and social/structural issues	3.75	5	3.54	3	7.29	8
2. Community perspectives	3.31	13	3.36	8	6.67	21
3. HIV stigma and adolescent development	3.80	2	2.96	14	6.76	16
4. Alignment of policy and culture	3.71	6	3.29	10	7.00	16
5. Academic and policy traditions	3.10	15	2.72	15	5.82	30
6. Workforce/workflow	4.01	1	3.38	7	7.39	8
7. Structure of implementation efforts	3.77	4	3.58	2	7.35	6
8. Appropriateness of care	3.69	7	3.51	4	7.20	11
9. Stakeholder alignment	3.61	8	3.14	12	6.75	20
10. Threshold conditions for treatment	3.79	3	3.66	1	7.45	4
11. Access to treatment	3.55	9	3.11	13	6.66	22
12. Stakeholder collaboration	3.18	14	3.20	11	6.38	25
13. Evidence communication	3.39	12	3.48	5	6.87	17
14. Program evaluation	3.46	10	3.33	9	6.79	19
15. Use of research evidence	3.42	11	3.38	6	6.88	17

Additional file 1 shows the bridging values and average importance and changeability ratings of all statements within each cluster, scored on a Likert scale of 0 to 5. The average importance ratings of each cluster ranged from 3.11 (5. Academic and Policy Traditions) to 4.01 (6. Workforce/Workflow). The average changeability ratings ranged from 2.72 (5. Academic and Policy Traditions) to 3.66 (10. Threshold Conditions for Treatment). The average importance and changeability ratings all fell above the Likert scale midpoint of 2.5.

Across the entire 15-cluster Concept Map, bridging values (listed in Additional file 1) were relatively high. The average bridging value was 0.41, indicating a trend toward statements to be sorted similarly across participants, with cluster averages ranging from 0.07 (13. Evidence Communication) to 0.80 (4. Alignment of Policy and Culture). Eight of the 15 clusters had average bridging values above 0.50, indicating a high level of conceptual overlap across the map. That is, the content of some clusters had some shared meaning with other clusters and thus requires greater care in interpretation compared to those with low bridging values.

Figure 3 shows the Pattern Match Map, including the correlation between each cluster’s average importance and changeability ratings. The overall correlation between the average importance and changeability ratings for the 15 clusters was *r* = 0.44. Figure 4 shows the Go Zone Map, with all 102 individual statements plotted by their average importance and changeability ratings.

## Discussion

The purpose of this study was to provide AHISA and other similarly focused stakeholders information on key gaps and opportunities for adolescent HIV programming and implementation research. We did this by identifying factors that hinder or facilitate the implementation of evidence-based prevention and intervention practices for adolescent HIV in sub-Saharan Africa. Through a mixed-method CM approach, we identified 15 distinct constructs that impact the implementation process (Table 2). The three clusters with the highest average importance ratings were as follows: 6. Workforce/Workflow (4.01), 3. HIV Stigma and Adolescent Development (3.80), and 10. Threshold Conditions for Treatment (3.79). The three clusters with the highest average changeability ratings were as follows: 10. Threshold Conditions for Treatment (3.66), 7. Structure of Implementation Efforts (3.58), and 1. Adolescent Engagement and Social/Structural Issues (3.54). It is notable that cluster 10, Threshold Conditions for Treatment, was rated high on both importance and changeability, suggesting that action items targeting this area may be the most effective and impactful in improving implementation efforts.

The clusters with the lowest changeability ratings were 11. Access to Treatment (3.11), 3. HIV Stigma and Adolescent Development (2.96), and 5. Academic and Policy Traditions (2.72). Cluster 3, HIV Stigma and Adolescent Development, showed the greatest discrepancy in importance and changeability ratings; while it was rated as highly important, it also received the second lowest changeability score. Policy-driven efforts may be crucial to impact change in these areas, and these clusters highlight areas of opportunity for policymakers and government funders to actively support and drive demonstrable change. For example, policies that increase funds for adolescent-focused health programs provide a direct avenue for expanding access to treatment.

The five statements with the highest changeability and importance ratings (shown in Additional file 1) were as follows: 87. Involvement of adolescents as stakeholders, 2. Lack of involvement of adolescents as their own change agents, 1. Adolescents lack knowledge about effective HIV prevention strategies such as PrEP, 58. Involve relevant community members in decision making, and 51. Building in-country capacity to deliver interventions. Taken together, these statements point to the need for greater involvement of community stakeholders, particularly adolescents, who are the target beneficiaries of these EBPs. As such, it may be important for implementers to design IS projects using community-based participatory methods. Funders may also support approaches such as participatory research and align funding mechanisms to facilitate more community-engaged approaches to implementation research and practice.

### Determinants and mechanisms placed in the EPIS framework constructs and phases

It is also important in implementation science to place work in an appropriate framework. The EPIS framework has been used in multiple studies in sub-Saharan Africa (e.g., Nigeria, Kenya, Sierra Leone) [13–15]. The EPIS framework is particularly useful in this context due to the overall constructs such as outer and inner contexts, but also the content of larger constructs such as the sociopolitical context represented in the outer context. Other implementation determinants, such as leadership, appear in outer and inner contexts throughout the four EPIS phases.

For example, there is almost always an interplay of outer system context and inner organizational context. The outer context has to do with the governmental or community characteristics, processes, and dynamics. The dynamics of outer context are important across more and less developed countries. For example, as in more industrialized countries, in LMICs, there is often a priority on certain health conditions and less emphasis on others. HIV is a critical public health issue with some issues represented in the EPIS outer context (e.g., population characteristics) and others in the inner context (e.g., clinicians in community clinics). However, other issues such as HIV-related stigma may limit engagement in services that are supported by governmental organizations. NGOs that provide HIV services may rely not only on in-country government funds, but also on research and service grants that can also focus on decreasing stigma. Bridging factors or processes (e.g., relational ties, formal arrangements, capital exchange, community-academic partnerships, collaborations, contracting) can link outer and inner contexts and serve as mechanisms by which outer context policies and funding are translated into action by inner context NGOs [15, 16, 26]. In the present study, the cluster dimensions clearly invoke EPIS constructs. For example, cluster 4 “Alignment of Policy and Culture” would reside in the outer context of a given country. On the other hand, cluster 6 “Workforce/Workflow” primarily resides in the inner context of organizations that deliver care. Cluster 9 “Stakeholder Alignment” can be considered a bridging factor. The higher the degree to which there is alignment in mission, vision, policy, and funding for EBPs, the more successful implementation efforts are likely to be [27]. In addition to determinants and mechanisms, various factors may fall into different EPIS phases. For example, cluster 13 would likely be best in the exploration phase, and cluster 15 “Use of Research Evidence” would naturally fall into the EPIS preparation and implementation phases. Cluster 14 “Program Evaluation” is important for understanding activities in the implementation phase but also important in the sustainment phase so that it is possible to understand if expected outcomes are being achieved.

### Parallels with PMTCT programming and research

The experiences and perspectives of different stakeholders may vary depending on their roles (e.g., policymaker, researcher, clinician, and patient) and the implementation setting. In 2012, the Fogarty International Center established an alliance of stakeholders focused on IS in the context of the prevention of mother-to-child transmission of HIV (PMTCT) [28]. Similar to AHISA, the PMTCT Alliance was comprised of stakeholders including PMTCT researchers, program implementers, and policymakers working in sub-Saharan Africa. In a CM analysis on determinants of PMTCT EBP implementation, this group identified 12 relevant clusters/domains, with “Governmental Commitment” and “Data Measurement and Collection” as the most highly ranked among the ratings for importance and changeability [29]. However, while ranked of the highest importance, “Health System Resources, Tracking, and Monitoring” ranked low on changeability (7th out of 12). This highlighted a disconnect between prioritization and action for PMTCT in sub-Saharan Africa and suggested a need for fundamental changes in government/funder financing policies and practice. In their scoping review, Ngidi et al. discussed three main categories of PMTCT EBPs in sub-Saharan Africa: health service delivery, health systems, and community level [30]. Their review was focused on the identification and description of EBPs rather than determinants of implementation. However, they highlighted EBP costs as potential barriers, while identifying implementer role clarifications, strengthening data quality/systems and EBP-program integration, and capacity-building for IS among healthcare workers as potential facilitators of effective EBP implementation [30]. Although implementation processes are often complex, methods such as CM can be used to summarize these experiences and perspectives across projects, phases, and system and organizational levels. For example, through a stakeholder-based, participatory, CM approach, Aarons et al. [29] identified key domains to be considered for PMTCT implementation research in sub-Saharan Africa.

There is a paucity of studies addressing barriers and facilitators in the implementation of EBPs for adolescent HIV services in sub-Saharan Africa. Most studies assess determinants from the user level in terms of service uptake and adherence [31–35], and not at the implementer/policymaker level in terms of EBP delivery [36, 37]. The few available studies on EBP implementation in adolescent HIV focus on site-level, point-of-care challenges, e.g., space challenges, inadequate management support and healthcare worker training, and not above-site government, policy, or strategy issues in implementation [36, 37]. Our findings are based on the experiences of AHISA stakeholders across multiple African countries, implementing different clinical interventions, and using different implementation strategies. They provide a robust and evidence-based IS foundation to guide researchers, implementers, and policymakers in future adolescent HIV projects. Taking stock of the barriers, bottlenecks, and available resources in each of these 15 domains could inform strategies to optimize implementation and services in the context of adolescent HIV prevention and treatment.

### Limitations

This study has a number of strengths, including a sufficient sample size for our CM approach, and representation across a number of disciplines, countries, and robust participant experience in implementation and HIV research. Methodologically, the CM approach was appropriate for this study; its virtual platform allowed for high-level engagement of our multi-disciplinary and geographically dispersed participant group throughout each study phase. However, some limitations should be noted. The primary role of most participants involved research, and there was less representation of those working in NGOs and policy settings. Furthermore, this study did not include certain stakeholders, such as patients and their families. Future research should include a wider representation of stakeholders, particularly those receiving services in the community, and with primary policymaker and implementer roles. Additionally, there was little variability in the importance and changeability ratings making it more difficult to prioritize some determinants and mechanisms over others. This is likely related to the homogeneity of participants, further emphasizing the need for future CM work to include more diverse groups of stakeholders.

This study included participants from 11 sub-Saharan African countries where AHISA member projects were being implemented. Although this broad representation bolsters the generalizability of our findings, it is likely that important country- or regional-level differences in implementation determinants and mechanisms exist. Differences in population demographics, adolescent HIV prevalence rates, or HIV programming capacities between countries may impact the emergence of implementation barriers or facilitators, as well as their importance and changeability. For example, changes to “Workforce/Workflow” factors may be more feasible in a country with a larger provider workforce and more funding for HIV prevention and intervention. As such, characteristics unique to one country in comparison to others in the sub-Saharan African region should be considered when applying these findings.

## Conclusions

Implementing EBPs is a complex endeavor, as is identifying and addressing the needs of adolescents with respect to HIV prevention and treatment. Robust context-specific knowledge is important in driving implementation of EBPs to address the most pressing health needs of target populations. It should specifically consider the importance and changeability of context-specific factors that impact EBP implementation. This study can provide guidance on factors to consider when designing implementation and sustainment strategies for adolescent HIV prevention and treatment in sub-Saharan Africa. Findings suggest that “Workforce/Workflow” and “HIV Stigma and Adolescent Development” were the most important factors affecting implementation, while “Threshold Conditions for Treatment” and “Structure of Implementation Efforts” were the most changeable factors. Further, placing these efforts in implementation frameworks can help to organize how importance and changeability are considered, staged, and planned for in the implementation process. The multi-disciplinary consideration of factors that impact implementation will likely have utility for other health conditions and in other contexts.

## Supplementary Information

**Additional file 1.** Bridging value and average importance and changeability ratings of all statements within each cluster.

## Data Availability

Not applicable

## Abbreviations

AHISA: Adolescent HIV Implementation Science Alliance
EBP: Evidence-based practice
EPIS: Exploration, Preparation, Implementation, Sustainment framework
CM: Concept mapping
HIV: Human immunodeficiency virus
IS: Implementation science
NGO: Non-governmental organization
PMTCT: Preventing mother-to-child HIV transmission
