# Correction to: Implementation determinants and mechanisms for the prevention and treatment of adolescent HIV in sub-Saharan Africa: concept mapping of the NIH Fogarty International Center Adolescent HIV Implementation Science Alliance (AHISA) initiative

**DOI:** 10.1186/s43058-021-00167-0

**Published:** 2021-07-07

**Authors:** Gregory A. Aarons, Kendal Reeder, Nadia A. Sam-Agudu, Susan Vorkoper, Rachel Sturke

**Affiliations:** 1 Department of Psychiatry, University of California San Diego, 9500 Gilman Drive (0812), La Jolla, CA 92093-0812 USA; 2 UC San Diego Dissemination and Implementation Science Center (UC San Diego ACTRI DISC), Altman Clinical and Translational Research Institute, La Jolla, CA 92093 USA; 3 Child and Adolescent Services Research Center, 3665 Kearny Villa Rd., Suite 200 N, San Diego, CA 92123 USA; 4 Institute of Human Virology and Department of Pediatrics, University of Maryland School of Medicine, 725 West Lombard Street, Baltimore, MD 21201 USA; 5 International Research Center of Excellence, Institute of Human Virology Nigeria, Plot 252 Herbert Macaulay Way, Abuja, Nigeria; 6 NIH Fogarty International Center, Center for Global Health Studies, Bethesda, MD USA

**Correction to: Implement Sci Commun 2, 53 (2021)**

**https://doi.org/10.1186/s43058-021-00156-3**

Following publication of the original article [1], it was reported that the following disclaimer was missing:

NIH Disclaimer: The content is solely the responsibility of the authors and does not necessarily represent the official views of the U.S. Department of Health and Human Services and the National Institutes of Health.

The original article has been updated to include this statement.
