# Supplementary material for: Implementation determinants and mechanisms for the prevention and treatment of adolescent HIV in sub-Saharan Africa: concept mapping of the NIH Fogarty International Center Adolescent HIV Implementation Science Alliance (AHISA) initiative
Source: Implement Sci Commun. 2021 May 22;2:53. doi: 10.1186/s43058-021-00156-3 (PMC8141156; doi:10.1186/s43058-021-00156-3)
Supplement: Supplementary file 1 — Additional file 1. Bridging value and average importance and changeability ratings of all statements within each cluster. [file 43058_2021_156_MOESM1_ESM.pdf]

Additional File 1. Bridging value and average importance and changeability ratings of all statements within each cluster

Cluster		Statement	Bridging Value	Average Importance Rating	Average Changeability Rating
**1. Adolescent Engagement and Social/Structural Issues**			**0.69**	**3.75**	**3.54**
	1	Adolescents lack knowledge about effective HIV prevention strategies such as PrEP	0.86	3.74	4.41
	2	Lack of involvement of adolescents as their own change agents	0.56	4.09	4.16
	11	Requiring parental consent for health services for adolescents under 16	0.74	3.97	3.22
	17	Religiosity and lack of belief in orthodox medicine	0.54	2.70	2.09
	58	Involve relevant community members in decision making	0.68	4.00	4.06
	87	Involvement of adolescents as stakeholders	0.84	4.27	4.44
	99	Cultural issues and taboos	0.57	3.52	2.42
**2. Community Perspectives**			**0.56**	**3.31**	**3.36**
	20	Community understanding of implementation challenges	0.61	3.24	3.72
	27	Patient advocacy groups	0.6	3.06	3.41
	71	Resistance to HIV prevention programmes by schools and communities	0.47	3.65	2.97
**3. HIV Stigma and Adolescent Development**			**0.37**	**3.80**	**2.96**
	4	Stigma around HIV	0.39	4.24	3.53
	32	Adolescence is vulnerable development stage	0.32	3.91	2.09
	44	Adolescent HIV status disclosure	0.39	3.85	3.59
	52	Stigma around adolescent sexuality	0.4	4.12	3.25
	76	Lack of caregiver support	0.42	3.21	3.22
	80	Parents/family acceptance that adolescents are sexually active	0.31	3.82	3.03
	94	Family status of the adolescent (e.g., orphan, parents)	0.35	3.47	1.97
**4. Alignment of Policy and Culture**			**0.8**	**3.71**	**3.29**
	9	Discrepancy between policy and cultural norms	0.65	3.56	3.28
	53	Discrepancy between policy and social norms	0.67	3.71	2.78
	85	Ethical challenges of involving person less than 18 years in research	1	3.85	3.13
	92	Lack of community participation in research	0.89	3.73	3.97

**5. Academic and Policy Traditions**		**0.54**	**3.10**	**2.72**
25	Funding for policy initiatives	0.81	3.71	2.97
36	Complexity of policy practitioner diplomacy when donors/funding is at stake	0.32	3.30	2.66
54	Hypotheses are demand driven (e.g., what is the NIH funding)	0.37	3.21	2.72
57	Funding not flexible if new evidence comes in	0.75	3.09	2.91
61	Diplomatic tradition	0.51	2.29	1.97
74	Policy tradition	0.51	2.73	2.52
101	Academics lack understanding of community implementation challenges	0.52	3.39	3.28
**6. Workforce/Workflow**		**0.44**	**4.01**	**3.38**
12	Potential sustainability of any program that is implemented	0.62	4.32	3.16
13	Cost of implementation	0.59	4.21	3.13
14	Lack of trained individuals to deliver programs	0.34	3.68	3.78
16	Providers' time/resources to provide high-quality care	0.32	4.21	3.13
51	Building in-country capacity to deliver interventions	0.49	4.21	3.81
79	Limited ability to track medical records across different health facilities	0.39	3.74	3.25
86	Lack of appropriately trained health care workers	0.33	3.73	3.41
**7. Structure of Implementation Efforts**		**0.54**	**3.77**	**3.58**
15	Leadership alignment across levels to create the climate for implementation	0.48	4.06	3.41
59	Prioritisation of specific interventions for individuals	0.56	3.18	3.32
70	Have programs with sound monitoring mechanisms	0.56	4.00	3.90
73	Limited ability to track medical records over time	0.48	3.59	3.47
88	Quality control	0.6	3.79	3.50
97	Multidisciplinary teams to work on implementation (e.g., MD, social work, managers)	0.55	4.00	3.87
**8. Appropriateness of Care**		**0.64**	**3.69**	**3.51**
30	Over-emphasis on treatment as prevention at the expense of primary prevention	0.63	3.21	3.00

63	Poor collaboration between school and health sectors	0.59	3.59	3.31
65	Ensuring programs are appropriately adapted for cultural relevance, feasibility, and acceptability	0.66	4.09	3.81
96	Consider the end-user needs	0.67	3.88	3.94
**9. Stakeholder Alignment**		**0.44**	**3.61**	**3.14**
50	In-country buy-in from key stakeholders to implement the program	0.44	4.09	3.56
67	Culture of not believing in evidence	0.56	3.09	2.81
75	Leadership is the real challenge	0.42	3.42	2.68
84	Policy makers/national government appreciation of adolescent HIV as a serious issue	0.34	3.85	3.50
**10. Threshold Conditions for Treatment**		**0.55**	**3.79**	**3.66**
18	Having an adolescent "champion" program (e.g. Center of Excellence)	0.7	3.71	4.19
24	Providers' negative attitudes towards youth seeking prevention services	0.4	3.94	3.53
33	Lack of targeted resources for adolescent HIV prevention programs	0.68	4.03	3.66
42	Transition from paediatric to adolescent and adult services	0.48	3.82	3.66
60	Family support facilitates uptake and adherence to treatment and prevention services	0.56	3.68	3.59
68	Treatment adherence	0.59	3.94	3.63
78	Use of incentives (e.g. covering part of transportation costs) helps treatment uptake in adolescents	0.6	3.24	3.13
91	Lack of "friendly" access to adolescent services	0.37	3.97	3.91
**11. Access to Treatment**		**0.34**	**3.55**	**3.11**
26	Relatively small numbers of adolescents in most care programs makes it difficult to incorporate adolescent-focused programs	0.4	2.97	2.47
31	Lack of confidentiality in health facilities	0.34	3.59	3.44
40	Long wait times at health facilities	0.31	3.67	3.09
49	Provider discomfort discussing sexuality with young people	0.34	4.00	3.25
77	Services are not convenient	0.32	3.50	3.32
**12. Stakeholder Collaboration**		**0.2**	**3.18**	**3.20**
3	Myth that there is a disconnect between research and policy and practitioners	0.09	2.76	3.09

5	Research business forum	0.33	2.18	2.46
6	Agendas are too much researcher driven	0.21	2.65	3.19
7	Influence funders to incorporate research evidence rather than ideological perspective	0.38	3.42	2.94
8	Have research, policy, and practice collaborate	0.12	4.21	3.63
10	Academic tradition	0.21	2.35	2.19
19	Lack of evidence based interventions targeting adolescents	0.24	3.59	3.78
34	No connections between researchers and policy makers	0.03	3.03	3.47
37	Lack of HIV prevention strategies with scientific evidence	0.43	3.03	3.38
66	Researchers communicating to the end user	0.17	3.76	3.84
83	Ask the right questions at the right time (for demographic shifts)	0.17	3.30	3.30
89	Time lag from research study to publication	0.12	3.45	2.81
93	Researchers working in silos	0.12	3.66	3.50
**13. Evidence Communication**		**0.07**	**3.39**	**3.48**
21	Identify level of evidence (low, medium, strong)	0.17	3.00	3.72
22	Use infographics to communicate research evidence	0.02	2.97	3.81
23	Clear definition of implementation research	0.07	2.91	3.63
28	Proactively identify research that may be useful in the future	0.05	3.44	3.50
29	Strong partnerships between program implementers and researchers	0.07	3.94	3.72
35	Increase the relevance of what and how researchers communicate	0.04	3.71	3.87
41	Policy and practice attend research dissemination	0.09	3.74	3.19
45	Be deliberate in how research evidence is reported	0.09	3.44	3.69
47	Getting consensus on what constitutes "research evidence"	0.11	3.32	2.87
55	Research to policy is a "broken telephone"	0	3.06	3.28
81	Difficult for policy to interpret academic publications	0.09	3.29	2.90
82	Communication between research and policy	0.02	3.85	3.56
**14. Program Evaluation**		**0.57**	**3.46**	**3.33**
38	Performance review meetings across offices and country	0.58	2.81	3.13
46	Leadership across levels	0.42	3.91	2.84
56	Evidence based implementation of youth friendly health services	0.74	3.76	3.56
62	Use program data for evaluation and decision making	0.51	3.71	3.59

64	Use CQI approaches (e.g., small tests of change)	0.63	3.12	3.52
**15. Use of Research Evidence**		**0.27**	**3.42**	**3.38**
39	Policy maker trained in what is evidence can be rolled out	0.25	3.39	3.44
43	Deciding what policy to implement	0.3	3.71	3.47
48	Policy must consider how the end-user will implement	0.33	3.76	3.69
69	Committee to address uptake of new evidence	0.2	3.03	3.13
72	Develop internal research capacity	0.31	3.68	3.84
90	Complexity of data (volume, velocity, variety)	0.22	3.52	2.97
95	Collaborators meet regularly (e.g., weekly) to consider existing and new evidence	0.21	3.09	3.00
98	Limited capacity to filter out bad from good research	0.29	3.00	3.09
100	The need to do evidence-based programming ("This is the decade of E = evidence.")	0.33	3.53	3.59
102	Lack of appreciation of evidence-based programming	0.29	3.47	3.53
